# EndoTime: non-categorical timing estimates for luteal endometrium

**DOI:** 10.1093/humrep/deac006

**Published:** 2022-01-29

**Authors:** Julia Lipecki, Andrew E Mitchell, Joanne Muter, Emma S Lucas, Komal Makwana, Katherine Fishwick, Joshua Odendaal, Amelia Hawkes, Pavle Vrljicak, Jan J Brosens, Sascha Ott

**Affiliations:** 1 School of Life Sciences, University of Warwick, Coventry, UK; 2 Warwick Medical School, University of Warwick, Coventry, UK; 3 Tommy’s National Centre for Miscarriage Research, University Hospitals Coventry and Warwickshire National Health Service Trust, Coventry, UK; 4 Bioinformatics RTP, Research Technology Platforms, University of Warwick, Coventry, UK

**Keywords:** luteal phase, endometrium, embryo implantation, recurrent pregnancy loss, computational biology

## Abstract

**STUDY QUESTION:**

Can the accuracy of timing of luteal phase endometrial biopsies based on urinary ovulation testing be improved by measuring the expression of a small number of genes and a continuous, non-categorical modelling approach?

**SUMMARY ANSWER:**

Measuring the expression levels of six genes (*IL2RB*, *IGFBP1*, *CXCL14*, *DPP4*, *GPX3* and *SLC15A2*) is sufficient to obtain substantially more accurate timing estimates and to assess the reliability of timing estimates for each sample.

**WHAT IS KNOWN ALREADY:**

Commercially available endometrial timing approaches based on gene expression require large gene sets and use a categorical approach that classifies samples as pre-receptive, receptive or post-receptive.

**STUDY DESIGN, SIZE, DURATION:**

Gene expression was measured by RTq-PCR in different sample sets, comprising a total of 664 endometrial biopsies obtained 4–12 days after a self-reported positive home ovulation test. A further 36 endometrial samples were profiled by RTq-PCR as well as RNA-sequencing.

**PARTICIPANTS/MATERIALS, SETTING, METHODS:**

A computational procedure, named ‘EndoTime’, was established that models the temporal profile of each gene and estimates the timing of each sample. Iterating these steps, temporal profiles are gradually refined as sample timings are being updated, and confidence in timing estimates is increased. After convergence, the method reports updated timing estimates for each sample while preserving the overall distribution of time points.

**MAIN RESULTS AND THE ROLE OF CHANCE:**

The Wilcoxon rank-sum test was used to confirm that ordering samples by EndoTime estimates yields sharper temporal expression profiles for held-out genes (not used when determining sample timings) than ordering the same expression values by patient-reported times (*GPX3*: *P *<* *0.005; *CXCL14*: *P *<* *2.7e−6; *DPP4*: *P *<* *3.7e−13). Pearson correlation between EndoTime estimates for the same sample set but based on RTq-PCR or RNA-sequencing data showed a high degree of congruency between the two (*P *=* *8.6e−10, *R*^2^ = 0.687). Estimated timings did not differ significantly between control subjects and patients with recurrent pregnancy loss or recurrent implantation failure (*P *>* *0.05).

**LARGE SCALE DATA:**

The RTq-PCR data files are available via the GitHub repository for the EndoTime software at https://github.com/AE-Mitchell/EndoTime, as is the code used for pre-processing of RTq-PCR data. The RNA-sequencing data are available on GEO (accession GSE180485).

**LIMITATIONS, REASONS FOR CAUTION:**

Timing estimates are informed by glandular gene expression and will only represent the temporal state of other endometrial cell types if in synchrony with the epithelium. Methods that estimate the day of ovulation are still required as these data are essential inputs in our method. Our approach, in its current iteration, performs batch correction such that larger sample batches impart greater accuracy to timing estimations. In theory, our method requires endometrial samples obtained at different days in the luteal phase. In practice, however, this is not a concern as timings based on urinary ovulation testing are associated with a sufficient level of noise to ensure that a variety of time points will be sampled.

**WIDER IMPLICATIONS OF THE FINDINGS:**

Our method is the first to assay the temporal state of luteal-phase endometrial samples on a continuous domain. It is freely available with fully shared data and open-source software. EndoTime enables accurate temporal profiling of any gene in luteal endometrial samples for a wide range of research applications and, potentially, clinical use.

**STUDY FUNDING/COMPETING INTEREST(S):**

This study was supported by a Wellcome Trust Investigator Award (Grant/Award Number: 212233/Z/18/Z) and the Tommy's National Miscarriage Research Centre. None of the authors have any competing interests. J.L. was funded by the Biotechnology and Biological Sciences Research Council (UK) through the Midlands Integrative Biology Training Partnership (MIBTP, BB/M01116X/1).

## Introduction

Menstruation is the defining characteristic of the endometrium in humans and higher primates, a trait otherwise found in only a handful of non-primate species (Bellofiore *et al.*, 2017). As a consequence of menstruation, the endometrium undergoes iterative cycles of tissue regeneration, rapid proliferation and differentiation, which cumulate in a transient window of implantation during the midluteal phase of the cycle. The window of implantation represents an inflection point in the cycle, after which the endometrium either breaks down or is transformed into the decidua of pregnancy, a specialized matrix that accommodates the placenta throughout gestation (Gellersen and Brosens, 2014). Endometrial cyclicity is driven by the rise and fall in ovarian oestrogen and progesterone production, triggering coordinated spatiotemporal gene expression changes in resident epithelial, stromal and vascular cells (Wang *et al.*, 2020). Furthermore, the midluteal window of implantation heralds the start of intense tissue remodelling, characterized not only by abrupt and dramatic changes in epithelial gene expression (Wang *et al.*, 2020), differentiation of stromal cells in pre-decidual cells (Lucas *et al.*, 2020) and angiogenesis (Demir *et al.*, 2010), but also by an influx of circulating innate immune cells (Strunz *et al.*, 2021), most prominently uterine natural killer cells (Brighton *et al.*, 2017) as well as non-haematopoietic bone marrow-derived progenitor cells (Diniz-da-Costa *et al.*, 2021).

It is widely accepted that pathological cues that interfere with the sequence of endometrial events leading to a functional implantation window causes reproductive failure, including recurrent implantation failure (RIF) and recurrent pregnancy loss (RPL) (Koot *et al.*, 2016; Lucas *et al.*, 2020). However, it has proven challenging to parse the precise underlying mechanisms. There are multiple challenges intrinsic to endometrial research, including heterogeneity in the cellular composition of endometrial biopsies (Suhorutshenko *et al.*, 2018), inherent inter-cycle variability in local immune cells (Brighton *et al.*, 2017) and, most prominently, the rapid temporal changes in gene expression across the luteal phase (Wang *et al.*, 2020). Accurate timing information is therefore critical in endometrial analysis (Devesa-Peiro *et al.*, 2021). While the average length of menstrual cycle is 28 days, there is considerable intra- and inter-individual variation (Soumpasis *et al.*, 2020). A pragmatic solution is to schedule biopsies relative to the pre-ovulatory LH surge (Tewary *et al.*, 2020). A prospective study on a small cohort of healthy women (n = 40) reported that the urinary LH surge occurs mostly within one day prior to ovulation, although the range was 4 days (Johnson *et al.*, 2015; Roos *et al.*, 2015). Furthermore, the rise in urinary pregnanediol-3-glucuronide, a progesterone metabolite, is more variable, occurring over a range of 5 days after ovulation (Johnson *et al.*, 2015; Roos *et al.*, 2015). Thus, while the timing of endometrial biopsies relative to clinical markers of ovulation is useful and convenient, it does not ensure comparable exposures to progesterone stimulation.

A complementary strategy is to infer timing by analysing the endometrial phenotype. Histological dating using the Noyes criteria was the foundational approach (Noyes *et al.*, 1950), but its accuracy has been brought into question (Coutifaris *et al.*, 2004; Murray *et al.*, 2004). Alternative methods for timing are based largely on detection of proteins, transcripts or microRNAs that mark the putative implantation window (Giudice and Saleh, 1995; Lessey, 1998; Develioglu *et al.*, 1999; Dubowy *et al.*, 2003; Kliman *et al.*, 2006; Aghajanova *et al.*, 2009; Sha *et al.*, 2011; Zhang *et al.*, 2012). In addition, several computational approaches for the prediction of the window of implantation are available commercially. The Win-test (Haouzi *et al.*, 2009, 2021), ERA (Díaz-Gimeno *et al.*, 2011; Ruiz-Alonso *et al.*, 2013) and ER Map/ER Grade (Enciso *et al.*, 2018) utilize gene panels of varying size (11, 238 and 40 genes, respectively) in order to categorize endometrial samples as pre-receptive, receptive, or post-receptive. However, these approaches not only offer limited temporal resolution but also risk misclassification of samples at the boundary of these time windows. At present, there are no cost-effective, validated methods to assess luteal phase endometrium in a continuous, time-dependent domain.

This study describes the development and validation of an expression-based assay that reflects time as a continuous measurement of days and hours, using a discrete set of temporal endometrial genes. For any given sample within a set, gene expression levels are used to define probability distributions based on the expression of all other samples in order to identify the most likely sample timing estimate with respect to the data of each gene, before being aggregated to provide a singular estimate. This process is then iterated, with each new series of estimated timings informing the next set of distributions until the process reaches convergence. Our method, termed EndoTime, is freely available as open-source software.

## Materials and methods

### Ethics

The study was approved by the NHS Research Ethics Committee, Hammersmith and Queen Charlotte’s & Chelsea Research Ethics Committee (1997/5065), and Tommy’s National Reproductive Health Biobank (REC reference: 18/WA/0356). All samples were obtained with written informed consent and in accordance with The Declaration of Helsinki (2000) guidelines.

### Endometrial sample collection

Endometrial biopsies were obtained from women attending the Implantation Clinic, a dedicated research clinic at University Hospitals Coventry and Warwickshire (UHCW) National Health Service Trust. Surplus tissues from endometrial biopsies obtained for diagnostic purposes were used for this study. Participants were instructed to use over-the-counter urinary ovulation tests and contacted the clinic on the day of a positive test or soon after. An endometrial biopsy was then scheduled 4–12 days after a positive test. The timing of endometrial biopsies was designated as LH+ (day), i.e. the number of days following a positive urinary ovulation test. Following transvaginal ultrasound assessment to exclude overt pelvic pathology, an endometrial biopsy was obtained using a Wallach Endocell™ endometrial sampler. All samples were immediately portioned with one part placed in RNAlater Stabilization Solution (Sigma-Aldrich, Dorset, UK) for a minimum of 24 h at 4°C before removal and storage at −80°C, one part snap frozen in liquid nitrogen, and one part fixed in 10% formalin for immunohistochemistry.

Two sets of RTq-PCR data were utilized in the development of EndoTime. Data Set I was used for model training, consisting of ΔCT values for 257 endometrial samples, with 96 provided by patients diagnosed with RPL, 81 by patients diagnosed with RIF and 80 acting as controls. Data Set II was applied in tandem with a trained model in order to provide timing estimates for 36 samples independently of patient-reported values. EndoTime was then applied to an independent data set, Data Set III, comprising of 407 LH-timed endometrial biopsies. Demographic information for all three data sets is presented in Supplementary Table SI. The number of samples at each timepoint (LH+) is shown in Supplementary Table SII. The sign for all ΔCT values in all three sets was first inverted in order to positively correlate with gene expression.

### RTq-PCR

Total RNA was extracted from RNAlater-protected endometrial biopsies using STAT-60 (AMS Biotechnology, Oxford, UK), according to the manufacturer’s instructions. Reverse transcription was performed from 1 µg RNA using the Quantitect Reverse Transcription Kit (QIAGEN, Manchester, UK) and cDNA was diluted to 10 ng/µl equivalent before use in qPCR. Amplification was performed on a Quant5 Real-Time PCR system (Applied Biosystems, Paisley, UK) in 10 µl reactions using 2× QuantiFast SYBR Green PCR Master Mix containing ROX dye (QIAGEN), with 300 nM each of forward and reverse primers. *L19* was used as a reference gene. Primer sequences of marker genes and the endometrial cell type(s) of expression are tabulated in Supplementary Table SIII.

### RNA-sequencing

RNA was purified using RNA STAT-60 (AMS Bio) according to manufacturer’s instructions and treated using Amplification Grade DNase I (Invitrogen) followed by ethanol precipitation and clean-up. Quality control, library preparation and sequencing were performed by the Wellcome Trust Centre for Human Genetics. Libraries were prepared using the Illumina TruSeq Stranded mRNA sample prep kit according to manufacturer’s instructions. Paired-end 75 bp sequencing was performed on Illumina HiSeq4000.

### Tissue imaging

Endometrial biopsies were fixed overnight in 10% neutral buffered formalin at 4°C and wax embedded in Surgipath Formula ‘R’ paraffin using the Shandon Excelsior ES Tissue processor (ThermoFisher). Tissues were sliced into 3 μM sections on a microtome and adhered to coverslips by overnight incubation at 60°C. Deparaffinization, antigen retrieval (pH 9), antibody staining, haematoxylin counter stain and 3,3’-diaminobenzidine colour development were fully automated in a Leica BondMax autostainer (Leica BioSystems). Tissue sections were stained for CD56 (a uNK cell-specific surface antigen) using a 1:200 dilution of concentrated CD56 antibody (NCL-L-CD56-504, Novocastra, Leica BioSystems). Stained slides were de-hydrated, cleared and cover-slipped in a Tissue-Tek Prisma Automated Slide Stainer, model 6134 (Sakura Flinetek Inc., CA, USA). Bright-field images were obtained on a Mirax Midi slide scanner using a 20× objective lens and opened in Panoramic Viewer v1.15.4 (3DHISTECH Ltd, Budapest, Hungary).

### Pre-processing of RTq-PCR data

Expression (ΔCT) values in individual samples in Data Sets I and III were normalized to a scale of zero to one per gene and then adjusted by a batch-specific additive constant as a modest batch effect correction, making mean expression values equal in each batch. Samples in Data Set II were processed for RTq-PCR analysis as a single batch.

### Pre-processing of rLH+ values

EndoTime modelling required that reported sample time points be converted from an ordinal to a continuous domain, a process that was undertaken in two steps. First, random noise sampled from a uniform distribution between −0.5 and 0.5 was added to each reported LH+ (rLH+) value. Second, samples were sorted in ascending order according to these updated timings, and the timings were smoothed using linear regression. This procedure allowed for each sample to be spaced evenly throughout the defined time course in a non-discrete manner, but close to its original rLH+ value, an approach that was considered robust in the presence of samples with unusually high or low reported timing values.

### EndoTime method

The approach for modelling via EndoTime relies upon an iteration of temporal gene expression profile refinement followed by the application of these refined profiles to estimate sample timings. Continuous rLH+ values generated during pre-processing were used to form initial expression profiles specific to each gene in the panel, which were then partitioned into windows of equal size (Supplementary Fig. S1). A normal distribution was used to model gene expression inside the time window with a mean inferred from samples inside the window and a weighted standard deviation based on the relative distance of points from the mean (Supplementary Fig. S2). Each window represented a singular time point derived from the median of binned reported time points. The first iteration utilized a bin size of 80 samples, which decreased by 10 with each successive iteration to a minimum of 20. Where temporal profiles deviate strongly from linearity, the normal distribution for data in a window could make for an inaccurate match of the data, but as window sizes are decreased deviations from linearity will become minor in later iterations of EndoTime.

Each sample within the data set was then assessed individually for its likely timing. The expression values for each marker gene within each window were used to generate a probability density curve based on the likelihood that the expression values observed in a sample were drawn from the distribution seen in the windowed data. This resulted in a set of six probability density curves being generated per sample, with each curve representing the results of attempting to estimate timing for the sample based on one gene alone, with associated curve maxima suggesting the time point with the greatest likelihood. Utilizing a shrinking bin size allowed for the first iteration of the process to filter out the majority of noise introduced by unreliable rLH+ into the data. Subsequent iterations refined estimations, while enforcing a minimum bin size ensured that the curves were smooth and presented a single clear maximum (Supplementary Fig. S3).

This process of generating six individual probability density curves also allowed for an assessment to be made regarding the congruency of their peak maxima and therefore the consistency with which each gene provided the same timing estimate. Synchronous samples were those wherein the six maxima all suggested a similar estimate, while asynchronous samples were those that presented conflicting estimations; an ‘asynchrony’ score was provided to each sample based on the standard deviation among all six maxima, which describes how coherently the aforementioned probability density curve-based approach provides a singular timing estimate.

Consolidating these six curves into a single curve by averaging their scaled densities allowed for the identification of a maximum in a single curve, which was used as the new time point estimation for the sample. A window-based approach was used when consolidating the six individual curves into one, with bin sizes equal to those used when generating the underlying reference profiles of panel gene expression. This process iterated until convergence, with each iteration undertaking both refinement of temporal profiles and time point estimation. The difference between the estimations provided by the preceding and current iteration was measured using the Euclidean distance and convergence was declared once the distance fell below 2.

During the modelling process, the absolute values of sample timings could deviate from the desired range as our method was primarily designed to optimize the correct order of samples, rather than retaining the original unit associated with timings. To ensure that EndoTime outputs are in line with original units, the raw timings obtained by modelling were converted following the last iteration such that the overall distribution of patient-reported LH times is approximately matched by the EndoTime output.

The six panel genes initially formed part of a set of 15 genes that were measured across the samples of Data Set I (Supplementary Table SIV), providing sufficient data to reconstruct the original temporal profiles in the presence of substantial noise. After establishing the EndoTime method, we gradually reduced this set of genes by successively removing the gene that least affected the Spearman correlation of sample ranks inferred with and without the gene. We stopped this process at six genes even though the correlation was still greater than 0.99 in order to be able to ascertain asynchrony scores with confidence.

### Pre-processing of RNA-seq data

RNA-seq libraries were mapped to the hg19 human genome assembly (2014) using Bowtie v. 2.2.3 (Langmead *et al.*, 2009), TopHat v.2.0.12 (Trapnell *et al.*, 2009) and Samtools v.0.1.19 (Li *et al.*, 2009) and reads mapped to features were counted via HTSeq v.0.6.1 (Anders *et al.*, 2015) prior to Transcripts Per Million (TPM) normalization.

In order to apply EndoTime to RNA-seq data, an approach was developed to convert read counts of EndoTime panel genes to pseudo-RTq-PCR data. TPM for each of the six timing panel genes were initially log2-transformed and then transformed to match the mean and standard deviation for each respective gene in the RTq-PCR data of Data Set I, with all processing performed using base functions in R v.4.0.2 (R Core Team, 2021).

### Statistical analyses

To assess the improvement in timing accuracy, we used a cross validation approach. Sample timings were estimated using EndoTime with a panel of only five genes, holding out the expression data for one gene. Expression data for the held-out gene was ordered (i) by patient-reported times (denoting this vector as *v_P_*), (ii) by EndoTime timing estimates (denoted as *v_E_*) and (iii) by expression level, in ascending order if the expression level of the held-out gene increases over time and descending order otherwise (denoted as *v_G_*). As patient-reported times are integer values with a unit of days, breaking ties needed to be resolved in order to compare directly against the other vectors. This was done by ordering samples of the same day in ascending or descending order according to the expression level of the held-out gene. We applied the Wilcoxon rank-sum test to check whether the absolute values of the differences *v_G—_v_P_* were greater than for *v_G—_v_E_* in a single-sided test. A significant *P*-value indicates that the order of samples provided by EndoTime is closer to the perfect order. In this setting, breaking the ties for patient-reported times as described above yields the largest *P*-value among all possible resolutions of ties, meaning that statistical significance may be under-stated but not over-stated with this approach as the *P*-value computed is an upper bound for the *P*-value that could be obtained if patient-reported times were more finely resolved. This process was repeated six times, holding out one panel gene at a time, and *P*-values Bonferroni-corrected for multiple testing.

RNA-seq data was examined via Principal Component Analysis in MATLAB following transformation of raw counts using the rlog function from the R library DESeq2 v.1.30.1 (Love *et al.*, 2014).

Patient demographics were assessed for normality via Shapiro–Wilk test. *P*-values for normally distributed data were then computed either via unpaired *t*-test (Data Set I) or *P*-values against control or 0 losses were computed via ordinary one-way ANOVA with Dunnett’s multiple comparisons test (Data Sets II and III). *P*-values for non-normally distributed data were calculated via Wilcoxon rank-sum test (Data Set I) or Kruskal–Wallis with Dunn’s multiple comparisons test (Data Sets II and III).

## Results

The EndoTime method was developed using two sample sets of luteal phase endometrial biopsies. Demographic information for both sample sets are presented in Supplementary Tables SI and SII. Data Set I consisted of 257 endometrial biopsies assayed by RTq-PCR in nine batches, out of which 96 were obtained from women with a history of RPL (defined here as 3 or more consecutive pregnancy losses), 81 were from women with repeated implantation failure (i.e. no positive pregnancy test following three or more transfers of day 5 blastocysts) and the remaining 80 biopsies were from control subjects. Refinement of the EndoTime gene panel resulted in the selection of six temporal marker genes, several of which are shared in the gene panels of existing computational methods for endometrial biopsy timing: all six genes can be found in the ERA panel and *GPX3* can be found in the ER-Map panel. The distribution of expression values for all six panel genes were found to be comparable between clinical cohorts (*P *>* *0.05, Wilcoxon rank-sum test, Supplementary Fig. S4). Data Set II consisted of 36 endometrial biopsies assayed by RTq-PCR and RNA-seq as a single batch. EndoTime was applied to 407 endometrial biopsies (Data Set III) to determine if the incidence of ‘mistimed’ or ‘out-of-phase’ samples relates to the recurrence risk of miscarriage. Supplementary Table SI provides demographic information on all three sample sets. The distribution of endometrial biopsies relative to the patient-reported positive urinary ovulation test is shown in Supplementary Table SII.

### The EndoTime method

**Figure 1.**
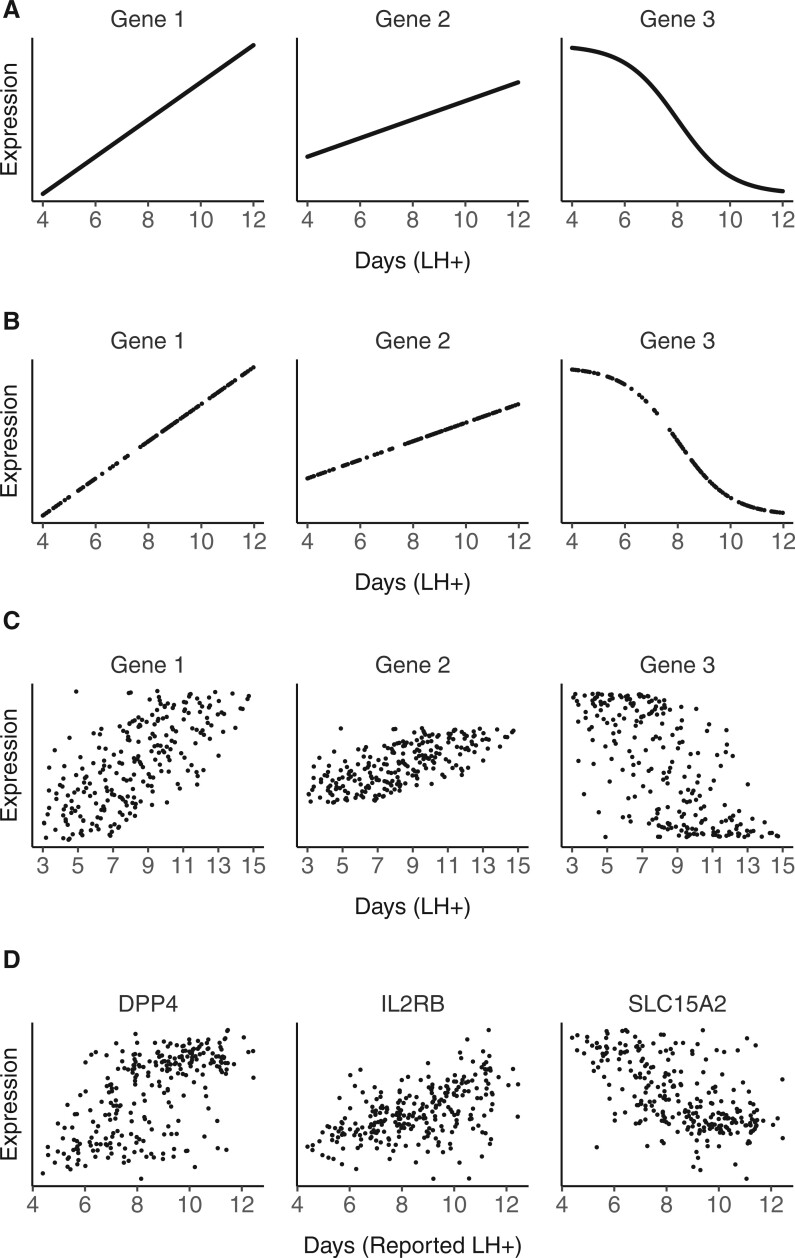
**Effect of sampling and noise on measured temporal profiles.** (**A**) Ideal expression curves for three artificial genes with infinite sampling density and without noise. (**B**) Simulated data as in A but with limited uniform sampling over the time axis more reflective of real-world biopsy availability. (**C**) Data simulated as in B with random noise added to time points (noise sampled from the normal distribution, mean = 0, SD = 2) to reflect uncertainty in reporting. (**D**) Expression measurements of three genes in clinical samples with patient-reported timings. The observed gene expression patterns are a good match for anticipated patterns simulated in C in terms of noise level and fuzzy appearance of temporal profiles. As original data are only resolved to full days, samples have been moved randomly on the time axis with an average displacement of 6 h (maximum of 12 h) to make data visualization more comparable with simulated data on a continuous domain.

Timing estimates of endometrial biopsies should ideally rely on temporal reference profiles of marker genes that span the entire luteal phase and are free of noise, as illustrated by synthetic data in Fig. 1A, generated using R via linear and logistic functions between hypothetical values for time and gene expression. In reality, only a limited number of biopsies can be sampled (Fig. 1B), and patient-reported days since a positive urinary ovulation test (rLH+) will be subject to a degree of error and noise as simulated in Fig. 1C, thus obscuring the true temporal expression patterns. We observed that the simulated data show a very similar pattern to real-world data (Fig. 1D), illustrating the practical relevance of this theoretical framework. EndoTime aims to minimize the impact of this source of noise by recovering the original expression patterns and thereby allowing for more accurate estimation of endometrial timing.

**Figure 2.**
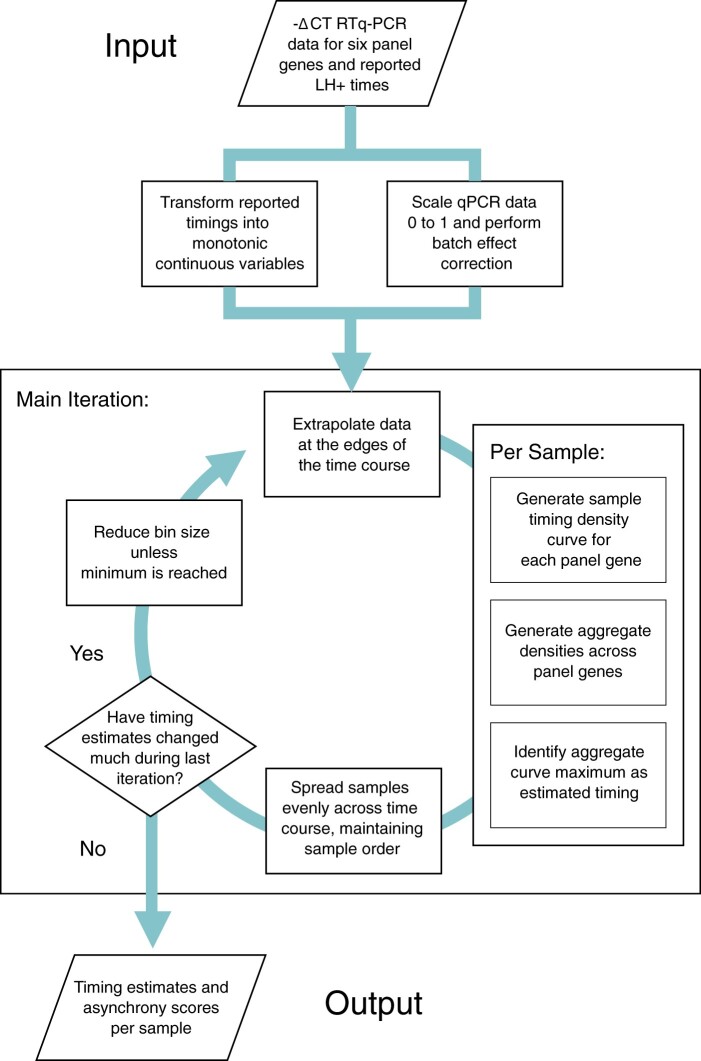
**Estimation of endometrial sample timings by EndoTime.** Pre-processing steps normalize and apply minor batch correction to –ΔCT qPCR gene expression data and transform reported sample timings, in LH+, into a continuous domain suitable for modelling. The bulk of the computation is an iterative process of binning data, generating an aggregate pseudo-density curve per sample, the maxima of which are selected as an estimate for the most likely sample timings, and assessment of the relative difference in sample estimates between one iteration and the preceding one. Once the difference in estimates falls below a predefined threshold value, diminishing returns are considered to have been reached and the modelling process ceases, returning the final sample timing estimates and an associated values for asynchrony, which reflect the degree to which all six panel genes agree upon estimates.

**Figure 3.**
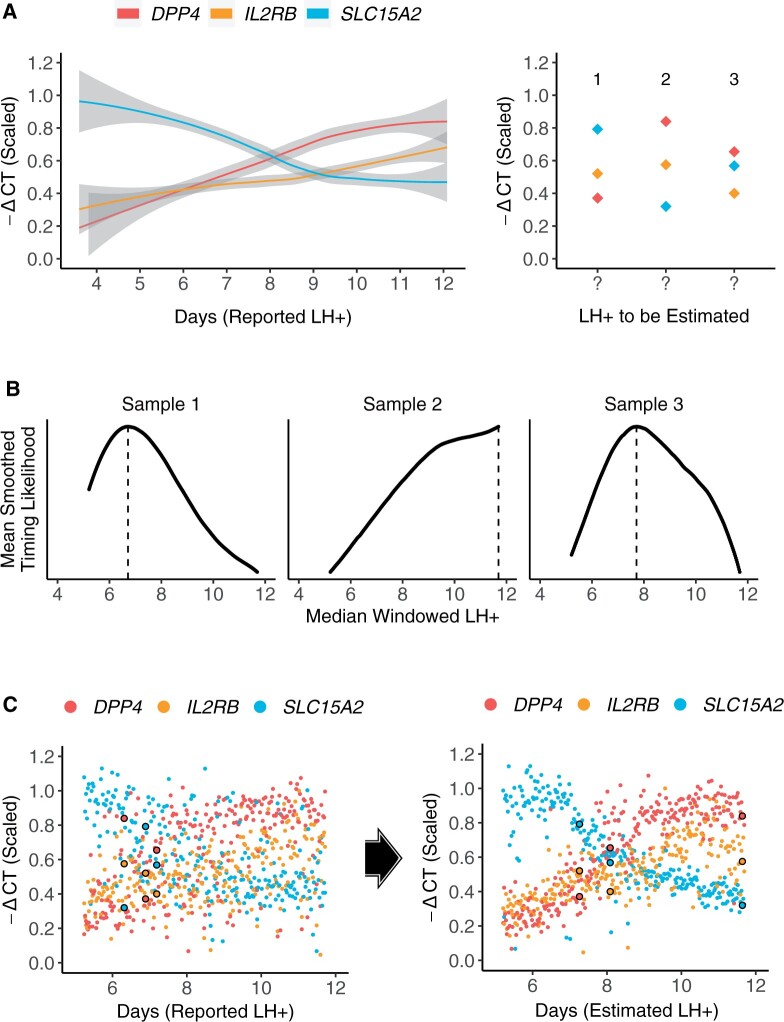
**Illustration of one iteration of the EndoTime modelling process.** (**A**) Computing temporal profiles. Left: Regression curves fit to expression data for three genes of the timing marker panel. Right: Expression values for three samples from the training data are the basis for re-evaluating timings of these samples. (**B**) Temporal profiles learned in A are used to improve time point estimates. For each sample, the likelihood of each time point is computed, with suggested sample timing represented by peak maxima for each sample. (**C**) Improved time point estimates provide improved temporal profiles. Left: Expression data arranged according to patient-reported LH+. Values for the three samples from A and B are circled. Right: Expression data re-arranged according to new time estimates obtained in B. Expression curves are visibly tighter and more distinct after just one iteration. EndoTime repeats this process until convergence.

Accomplishing this goal requires us to solve a Chicken and Egg problem: inferring the correct time point for a given biopsy requires accurate reference expression profiles, but recovery of these profiles relies on accurately timed biopsies. An overview of our approach to solving this problem can be seen in Fig. 2. This is an iterative approach, using the initial rLH+ time points to model expression profiles while accounting for uncertainty (Fig. 3A), then updating biopsy timings for all samples based on the modelled reference profiles (Fig. 3B). These two steps are iterated, with reference profiles gradually becoming less noisy as timing estimates are improved in a stepwise manner (Fig. 3C). The process is repeated until convergence, defined as a minimal overall change of sample timings from one iteration to the next.

Modelling temporal expression profiles is achieved using a window-based approach that considers samples in individual segments of the time domain and modelling their mean and variance as a normal distribution (Supplementary Fig. S1). The size of the windows is gradually decreased from iteration to iteration as sharper temporal profiles allow for a more detailed model of the reference profiles. The position of samples inside a window is considered when computing the means such that samples near the centre of the window have stronger influence than samples near the edge (Supplementary Fig. S2).

The modelled temporal profiles (Fig. 3A) are used to compute probability density functions for each sample and each marker gene, which show how likely each time point is for the given sample as judged by the reference profile of a single marker gene. Joint probability density functions are then computed, generated by a similar process of scaling and binning the density functions for all six marker genes per sample, followed by calculating the average density within each bin. The result is a singular ‘pseudo’-density function for each sample showing likelihood of sample timing based on the reference profiles for all marker genes (Fig. 3B). The maxima of these functions are then identified for each sample, which provide maximum likelihood estimates for the most appropriate sample timing. The iterated process of updating the reference profiles and updating sample timings gradually refines the reference profiles and increases the certainty in timing estimates (Fig. 3C, Supplementary Fig. S3).

### Validation of EndoTime method

**Figure 4.**
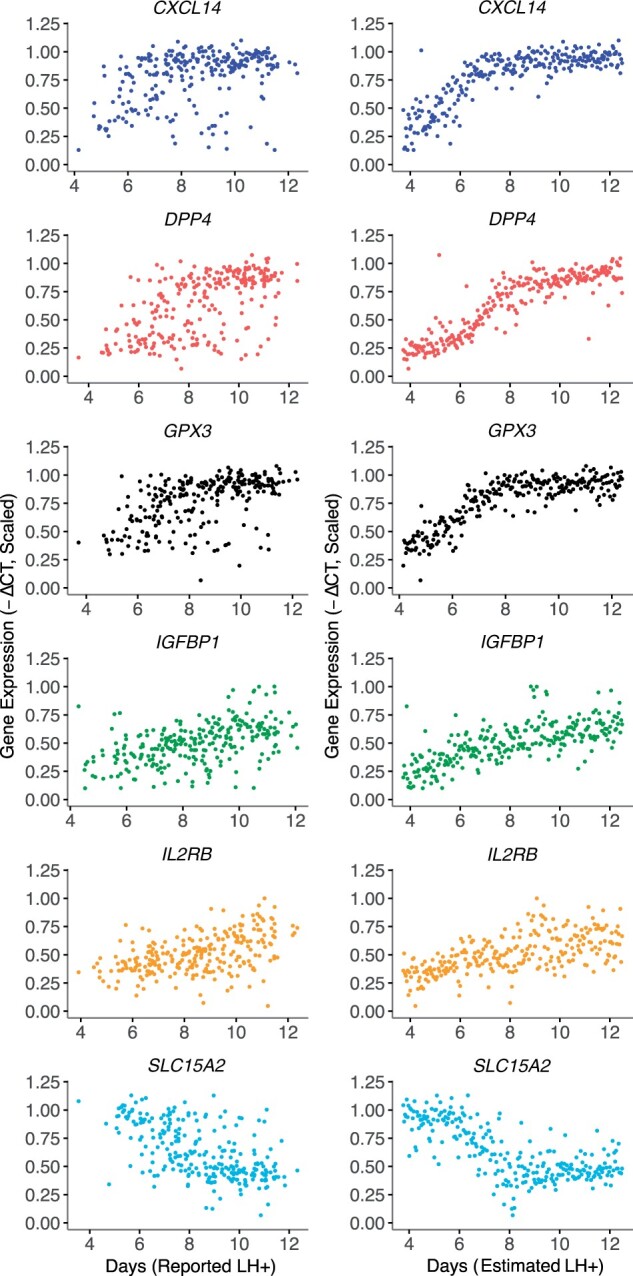
**Method validation by leave-one-out approach.** Left: Expression measurements for panel genes plotted using time points reported by patients. As original data are only resolved to full days, samples have been moved randomly on the time axis with an average displacement of 6 h (maximum of 12 h) to make data visualization more comparable with EndoTime estimates on continuous domain. Right: Temporal profile of each gene after using data of the other five genes to obtain timing estimates for all samples. Substantially sharper profiles show that EndoTime reveals the true order of samples more accurately than clinical records.

We applied the EndoTime method on Data Set I, comprising of 257 luteal endometrial samples. We used a leave-one-out cross-validation approach for the set of marker genes used, inferring timings based on five genes while not using the expression data of the held-out, sixth gene. We hypothesized that EndoTime estimates will yield sharper, less noisy temporal profiles for temporally regulated genes. If samples were ordered merely to fit the data of five genes without inferring the true order of samples, then the temporal profile for the held-out gene would not improve. This process was repeated six times, holding out one gene at a time. We found that the temporal profile of each held-out gene became tighter after EndoTime with expression values, deviating less from the temporal trajectory when compared to profiles plotted using patient-reported times (Fig. 4, right and left panels, respectively). This effect was particularly pronounced for *CXCL14*, *DPP4*, and *GPX3*. The Wilcoxon rank-sum test was used to confirm that the improvement in temporal expression profiles for three held-out genes was statistically significant (see Materials and methods; *GPX3*: *P *<* *0.005; *CXCL14*: *P *<* *2.7e−6; *DPP4*: *P *<* *3.7e−13). The other genes, though visually appearing tighter, did not test significantly under the conservative testing our approach, which resolves breaking ties for patient-reported times in a way that maximizes *P*-values (see Materials and methods). These genes may also be less tightly regulated in the luteal phase. We concluded that EndoTime arranged samples on the time axis in a biologically more accurate manner than patient-reported times.

### Detecting asynchronous samples

**Figure 5.**
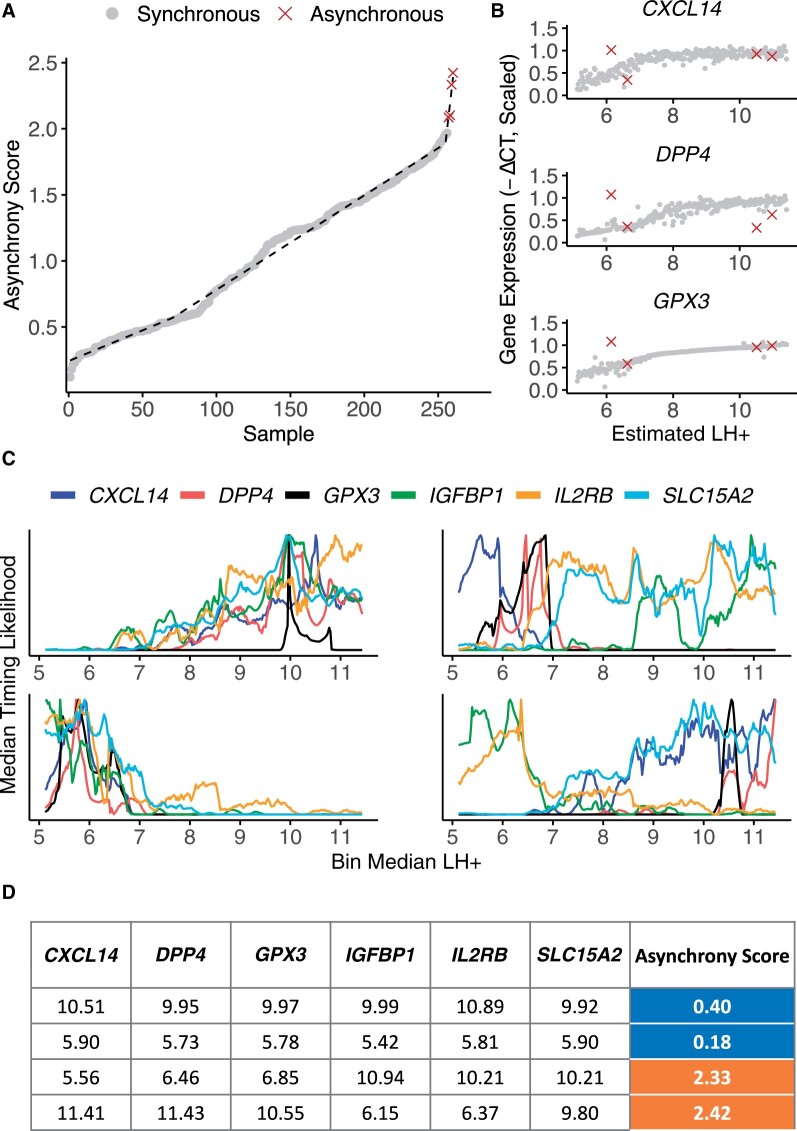
**Quantification of sample asynchrony based on consistency between panel genes.** (**A**) Samples are ranked according to their asynchrony score. Breakpoint of segmented linear model designates cut-off point for outliers. (**B**) Gene expression profiles for three timing panel genes following modelling, with outlying asynchronous samples highlighted. Each sample deemed asynchronous shows discrepant expression values for at least one gene. (**C**) Timing likelihood for all panel genes for two synchronous samples (left, top and bottom) and two asynchronous samples (right, top and bottom). Synchronous samples exhibit curves with maxima conforming towards a singular predicted time point, while asynchronous samples exhibit contradictory maxima. (**D**) Time point estimates based on maxima for each panel gene for samples shown in C.

While the order of samples computed by EndoTime reduces the variability in temporal profiles substantially, some individual samples appear to be outliers. We queried if it was possible to assess the reliability of estimates on a per-sample basis to enable the automatic detection of the least reliable samples. As EndoTime computes probability distributions of timing for each marker gene individually before aggregation, the procedure could be compared to a voting scheme where each marker gene has one vote, enabling an assessment of consistency among marker genes. We formulated a score to measure asynchrony between timing estimates based on individual marker genes (Fig. 5A). Samples with a high asynchrony score show large discrepancies between marker genes and account for the most outlying samples (Fig. 5B and C, right panel; and Fig. 5D, bottom panel). By contrast, synchronous samples show consistency among marker genes (Fig. 5C, left panel; Fig. 5D, top panel), and a good fit to the temporal profile (Fig. 5B). Thus, EndoTime’s asynchrony score can automatically inform the user about unreliable estimates, which may either be due to noise in experimental measurement for the affected samples or reflect asynchronous gene expression in the tissue. The user may decide to remove such samples from the analysis and refine further the temporal profiles and timing estimates for the remaining samples or, alternatively, repeat the cDNA conversion and RTq-PCR assay of samples deemed asynchronous.

### EndoTime can be applied to RNA-seq data

Using EndoTime analysis of the 257 biopsies in Data Set I yielded refined gene expression profiles, arranged according to the estimated timings. These profiles can subsequently be utilized alongside new sample sets, an application that is particularly useful if these new sets are not large enough to obtain detailed reference profiles, or if patient-reported timings are not available, which are necessary to initiate the training process.

**Figure 6.**
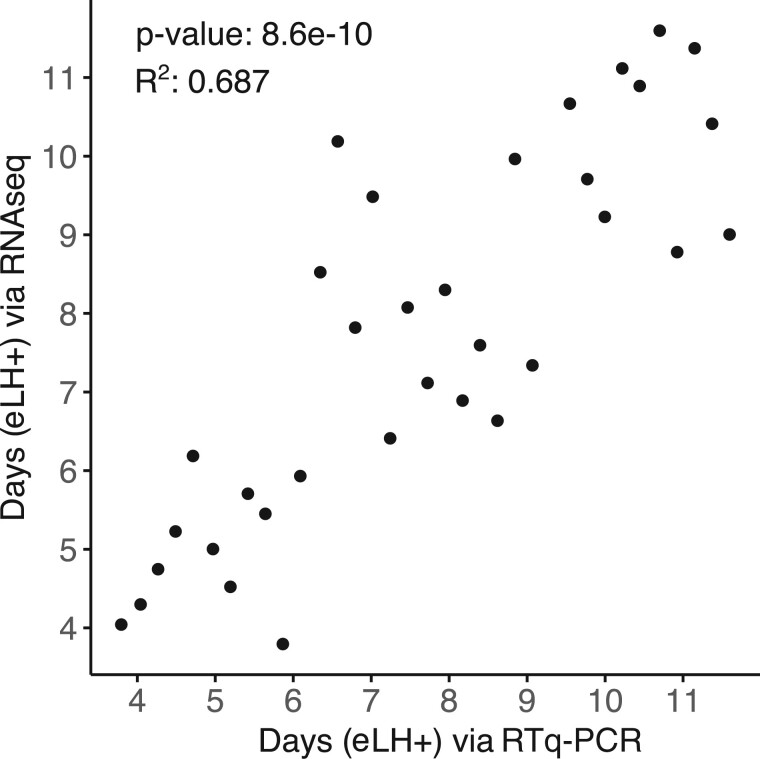
**Correlation of predicted time points from RTq-PCR data versus predictions from RNA-seq data of six panel genes.** eLH+, estimated LH+.

Data Set II consisted of 36 endometrial biopsies for which RTq-PCR as well as RNA-seq data across 33 329 genes was obtained as well as RTq-PCR data for the EndoTime gene panel. We used this set to assess if estimates derived from RTq-PCR data would yield comparable results when EndoTime is applied to measurements of the same six marker genes by RNA-sequencing. This necessitated normalizing the RNA-seq read counts to make these comparable to the normalized RTq-PCR values in terms of means and variances. As reference profiles were fixed by the modelling exercise for both data types, EndoTime was applied only to carry out a single estimation step for sample timings without updating the temporal profiles. Figure 6 demonstrates that RTq-PCR and RNA-seq time estimates are highly correlated (*P *=* *8.6e−10, *R*^2^ = 0.687). We concluded that meaningful EndoTime estimates can be obtained from RNA-seq data even if there is not enough data to re-train reference profiles.

### Inaccuracy of reported LH surge times

A fundamental concern towards reliance upon patient-reported timings provided with clinical biopsies is the potential for inaccuracy. The endometrium is intrinsically dynamic and mistimed samples could confound the diagnosis of underlying pathologies. Histological approaches can provide insights into biopsy timing but require additional processing of samples and appropriate expertise.

**Figure 7.**
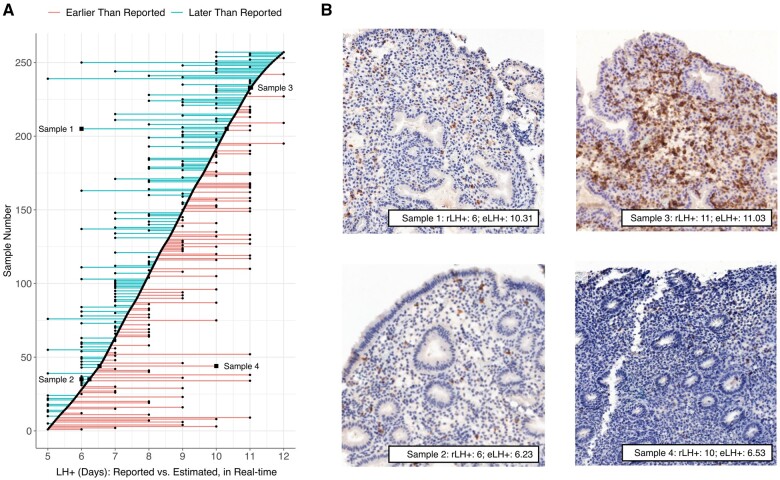
**Identification of mistimed samples.** (**A**) Samples shown in order identified by EndoTime (*y*-axis). EndoTime times shown as smooth curve. Deviations of reported timings from EndoTime timings shown as coloured horizontal lines. (**B**) Bright-field imaging with staining by the uNK marker CD56 for four samples. These images can be used to verify the progress of tissue development as earlier time points are associated with simple and tubular glands, while corkscrew-shaped glands are associated with biopsies donated during later time points. Samples 2 and 4 appear to be early samples, while Sample 1 and 3 are late samples. EndoTime estimates are consistent with this. Reported timings agree for Sample 2 and 3 but are discrepant for Sample 1 and 4 by about four days. eLH+, estimated LH+.

EndoTime analysis of the 257 biopsies in Data Set I revealed a mean difference between reported and estimated LH timing of 1.29 days, with 48 samples showing an estimated deviance of more than two days and 19 samples a deviance of more than three days. One biopsy was estimated to be 6.22 days later than the rLH+ value. The likelihood of mistiming appeared to be broadly independent of the temporal state of the tissue (Fig. 7A), with deviations occurring throughout the luteal phase. This disparity was also seen upon comparison of patient-reported timings with histological analysis of the tissue samples, the latter of which were congruent with the predictions provided by EndoTime (Fig. 7B).

### EndoTime captures primary source of transcriptomic variability in endometrium

**Figure 8.**
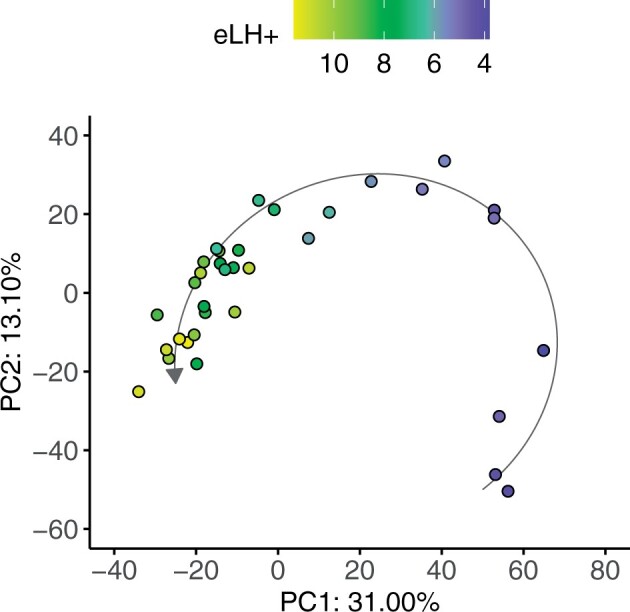
**EndoTime estimates capture largest single source of variability in endometrial transcriptomes.** PCA performed on 33 329-dimensional RNA-seq data. Colours indicate EndoTime timings inferred from just six genes which are consistent with sample positions in PC 1 and 2 which capture 44.1% of transcriptomic variation. eLH+, estimated LH+.

Appraisal of the influence of time on transcriptomic variability in comparison to other potential sources of variation, such as interpatient variability, was achieved by performing principal component analysis on the RNA-seq data in Data Set II. The two principal components that explained the largest percentage of variance overlaid RTq-PCR-based EndoTime estimations (Fig. 8), implying that at least 44.1% of variance among 33 329 genes measured can be explained by temporal fluctuations as measured accurately using just the six genes in the EndoTime panel. We conclude that EndoTime captures the primary parameter underlying transcriptomic variability in endometrial biopsies obtained during the luteal phase of the menstrual cycle.

**Figure 9.**
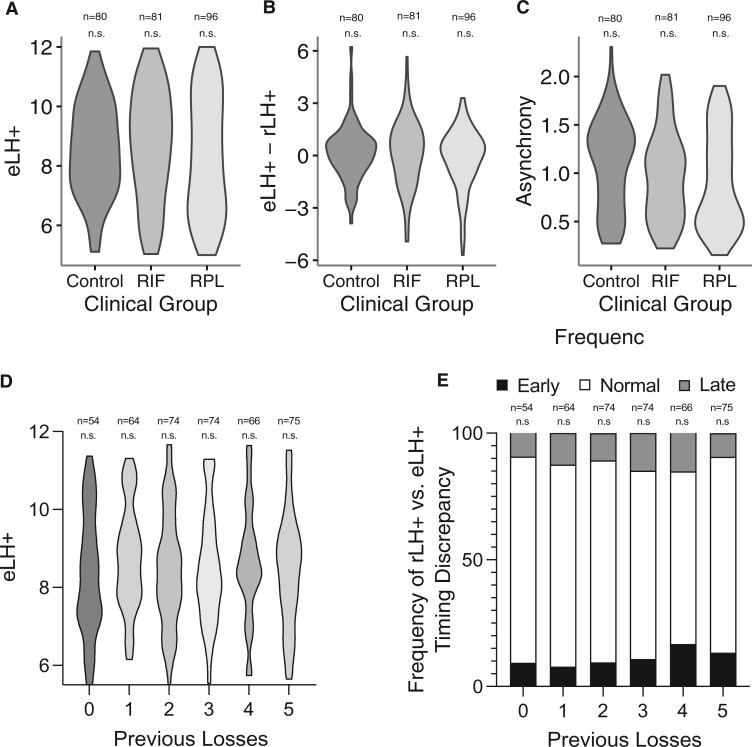
**Comparisons of EndoTime estimates across clinical groups.** (**A**) Distributions of EndoTime timing estimates (eLH+) between Control, RIF and RPL groups within Data Set I (*P *>* *0.05, Wilcoxon rank-sum test). (**B**) Distributions of the difference between reported LH+ (rLH+) and eLH+ between Control, RIF and RPL groups within Data Set I (*P *>* *0.05, Wilcoxon rank-sum test). (**C**) Distributions of gene asynchrony across Control, RIF and RPL groups within Data Set I (*P *>* *0.05, Wilcoxon rank-sum test with Bonferroni multiple testing correction). (**D**) Distribution of eLH+ according to number of historical pregnancy losses within Data Set III (*P *>* *0.05, ordinary one-way ANOVA with Dunnett’s multiple comparisons test). (**E**) Frequency of biopsies with reported timings that are either earlier (‘Early’), later (‘Late’) or within ±1.5 days of the eLH+ in function of the number of previous pregnancy losses (*P *>* *0.05, Chi-squared test). eLH+, estimated LH+; n.s., non-significant.

A shift in EndoTime between patient groups could indicate a role for ‘out-of-phase’ endometrium in driving reproductive failure. To test whether the clinical phenotype impacts on the performance of EndoTime, we first examined the expression levels of our six panel genes in control, RIF and RPL patients in Data Set I. As shown in Supplementary Fig. S4, the distribution of these genes was comparable between the clinical groups (*P *>* *0.05, Wilcoxon rank-sum test with Bonferroni multiple testing correction). Likewise, with EndoTime estimates, the difference between reported and estimated LH time and the asynchrony scores also did not differ between the groups (Fig. 9A–C). To validate these findings further, we applied EndoTime to Data Set III, which comprised 407 endometrial biopsies from women with a history of 0–5 miscarriages. Each miscarriage increases the risk of further pregnancy loss by ∼10%, independent of maternal age (Magnus *et al.*, 2019; Coomarasamy *et al.*, 2020; Kolte *et al.*, 2021). Hence, this sample set enabled testing whether the prevalence of ‘mistimed’ or ‘out of phase’ samples increases in function of the recurrence risk of miscarriage. Again, we found no evidence that EndoTime estimates are impacted by the likelihood of reproductive failure (Fig. 9D). As shown in Fig. 9E, neither the frequency of ‘early’ nor ‘late’ endometrial biopsies was affected by the number of previous pregnancy losses. EndoTime was also not influenced by either age or BMI (Supplementary Fig. S5).

## Discussion

EndoTime utilizes the transcriptomic profiles of an informative panel of genes to obtain temporal estimates in a continuous domain, rather than making a categorical classification. This avoids misclassifications that are likely when samples are close to the temporal threshold between different categorical phases of the cycle and increases the resolution of temporal analyses. Given that two of the existing, transcriptomics-based methods partition samples into just three categories, a misclassification into the neighbouring category implies substantially altered biological interpretation. We have shown the accuracy of EndoTime by leave-one-out validation, which involves removing one panel gene at a time and assessing the sharpening of the temporal profile of the held-out gene. In all cases, the results were comparable to those when using the entire panel, with only minimal increased noise in the estimated timings.

The combined advantages of measuring only six genes alongside freely available software mean that EndoTime minimizes the obstacles for wide adoption. EndoTime enables any measurements obtained from endometrial biopsies to be interpreted in relation to precise sample timing, thereby revealing the true temporal patterns much more accurately.

Importantly, the model training is part of the EndoTime software, enabling the application of EndoTime in other settings, for example with modified sets of panel genes or in different patient cohorts. In fact, EndoTime may be applicable to other tissues and other biological processes if the panel genes are chosen accordingly. Temporal patterns in the current panel genes are limited to monotonic shapes, either continuously increasing (five genes) or continuously decreasing (one gene) as these are the patterns found for most temporally variable genes in this tissue. Monotonic shapes are most informative as each expression level is only seen once across time, but the EndoTime methodology can be used for any temporal patterns. We believe that EndoTime has a range of applications in research settings as well as broad potential for clinical application as well.

EndoTime provides a good degree of transparency to the user, with each panel gene contributing its own estimate of sample timing, which are then aggregated in a single final time estimate. Estimates based on individual genes that appear inconsistent are reported to the user as asynchrony between panel genes, providing a measure of reliability and highlighting estimates with low confidence. Transcriptomic measurements in an individual biopsy sample can be plotted against the normal temporal profiles identified by EndoTime, providing a direct visual representation of the evidence for synchrony or asynchrony. Asynchrony may arise due to both technical errors and/or biological determinants. Although there was no observable correlation between timing errors and any of the three clinical groups that comprise Data Set I, future work could further investigate correlations of asynchrony with reproductive pathologies in larger data sets. Notably in this study, the concept of a biopsy being considered as ‘asynchronous’ relates to the relative congruence of all panel genes towards a singular estimated timepoint (Sebastian-Leon *et al.*, 2018).

EndoTime is able to provide timing estimations of greater accuracy as the size of contributing batches increases due to improved batch effect correction in the underlying transcriptomic data used for modelling. Samples in this study were obtained exclusively between 2 and 6 p.m., which limits the degree to which timing estimations might be influenced by fluctuations imposed by the circadian clock, such as those associated with *PER2* (Uchikawa *et al.*, 2011; Muter *et al.*, 2015). EndoTime’s accuracy might be improved via addition of an endometrial circadian gene to the panel and subsequent adjustments to the model should allow for greater timing resolution accounts for these daily rhythms. Of the six marker genes utilized, four are notably associated with the epithelium, implying that EndoTime estimates are mostly informed by the epithelial compartment of the endometrium.

By transforming RNA-seq measurements to match the distribution of RTq-PCR data prior to modelling via EndoTime, estimates can be obtained that are highly congruent. This conclusion was supported further upon projecting estimated timings over the principal component analysis of RNA-seq data, showing that over 44% of transcriptomic variance between samples can be explained as temporal fluctuations in gene expression. This offers the possibility of applying EndoTime to the transformation and timing estimation of endometrial RNA-seq data. This should broaden the application of EndoTime and identify additional temporally sensitive genes that might further improve the gene panel in the future. This may also provide a foundation for dissecting normal temporal changes from changes related to patient cohorts. In addition, it creates potential for developing methods for adjusting the timing of RNA-seq data sets computationally to make these more comparable across patient cohorts.

In summary, EndoTime is a novel open access software which advances the process of timing luteal phase endometrial biopsies along a continuous scale, presenting opportunities for further improvements in terms of its generalization across the entire endometrium. EndoTime’s application to a wider range of transcriptomic measurements and its timing resolution presents potentially far-reaching research and clinical applications.

## Data availability

The RNA-seq data are available in the Gene Expression Omnibus under accession GSE180485. The RTq-PCR data are available in the GitHub repository for the EndoTime software at https://www.github.com/AE-Mitchell/EndoTime.

## Supplementary Material

deac006_Supplementary_Figure_S1

deac006_Supplementary_Figure_S2

deac006_Supplementary_Figure_S3

deac006_Supplementary_Figure_S4

deac006_Supplementary_Figure_S5

deac006_Supplementary_Table_S1

deac006_Supplementary_Table_S2

deac006_Supplementary_Table_S3

deac006_Supplementary_Table_S4
