# Supplementary material for: EndoTime: non-categorical timing estimates for luteal endometrium
Source: Hum Reprod. 2022 Jan 29;37(4):747–61. doi: 10.1093/humrep/deac006 (PMC8971653; doi:10.1093/humrep/deac006)
Supplement: deac006_Supplementary_Figure_S1 [file deac006_supplementary_figure_s1.pdf]

Human Reproduction, pp. 1–1, 2022
https://doi.org/10.1093/humrep/deac006

human reproduction

SUPPLEMENTARY DATA

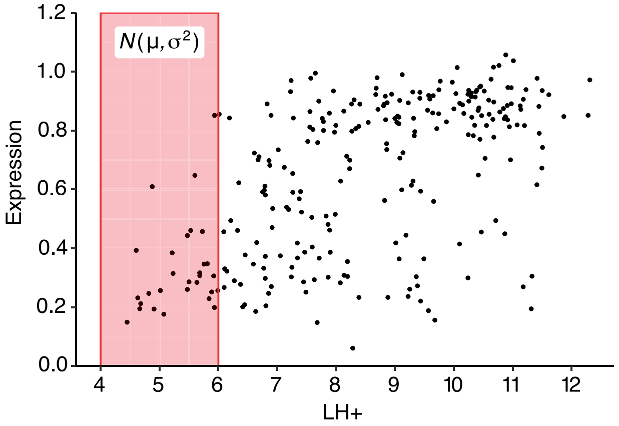

Supplementary Figure S1. Moving window estimates of expression distribution. Samples are ordered based on current estimates of endometrial timing. Expression at each time point is modelled as a normal distribution. A window (red shading) is placed around a given time point, mean and variance are estimated using only expression levels of samples inside the window. This is repeated for all window positions along the time axis.
