# Supplementary material for: EndoTime: non-categorical timing estimates for luteal endometrium
Source: Hum Reprod. 2022 Jan 29;37(4):747–61. doi: 10.1093/humrep/deac006 (PMC8971653; doi:10.1093/humrep/deac006)
Supplement: deac006_Supplementary_Figure_S2 [file deac006_supplementary_figure_s2.pdf]

**Human Reproduction, pp. 1–1, 2022**
https://doi.org/10.1093/humrep/deac006

human
reproduction

## SUPPLEMENTARY DATA

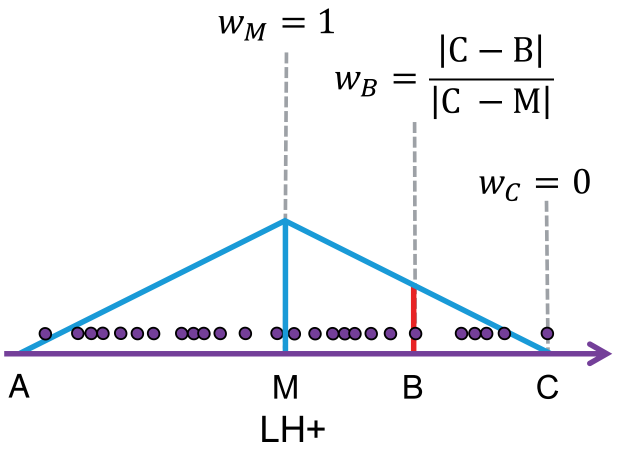


**Supplementary Figure S2. Weighted estimate of standard deviation.** *X* axis represents a single window; time estimates for samples inside the window are indicated by circles. Each sample contributes to the SD in dependence of proximity to the middle of the window, with closer samples contributing more. W: weight applied at relevant points (A, M, B and C), A: left-hand-side of window (weight of 0), M: middle position of the window (weight of 1), C: right-hand-side end of window (weight of 0), B: position of one sample, computation of weight for this sample is illustrated (weight between 0 and 1). Computation is shown for point B, which is located at a value of X larger than that of M. For hypothetical points smaller than M, this computation is mirrored and utilizes the left-hand-side end of the window with point A assigned a weight of 0 in place of point C.
