# Supplementary material for: EndoTime: non-categorical timing estimates for luteal endometrium
Source: Hum Reprod. 2022 Jan 29;37(4):747–61. doi: 10.1093/humrep/deac006 (PMC8971653; doi:10.1093/humrep/deac006)
Supplement: deac006_Supplementary_Figure_S3 [file deac006_supplementary_figure_s3.pdf]

Human Reproduction, pp. 1–1, 2022
https://doi.org/10.1093/humrep/deac006

human reproduction

SUPPLEMENTARY DATA

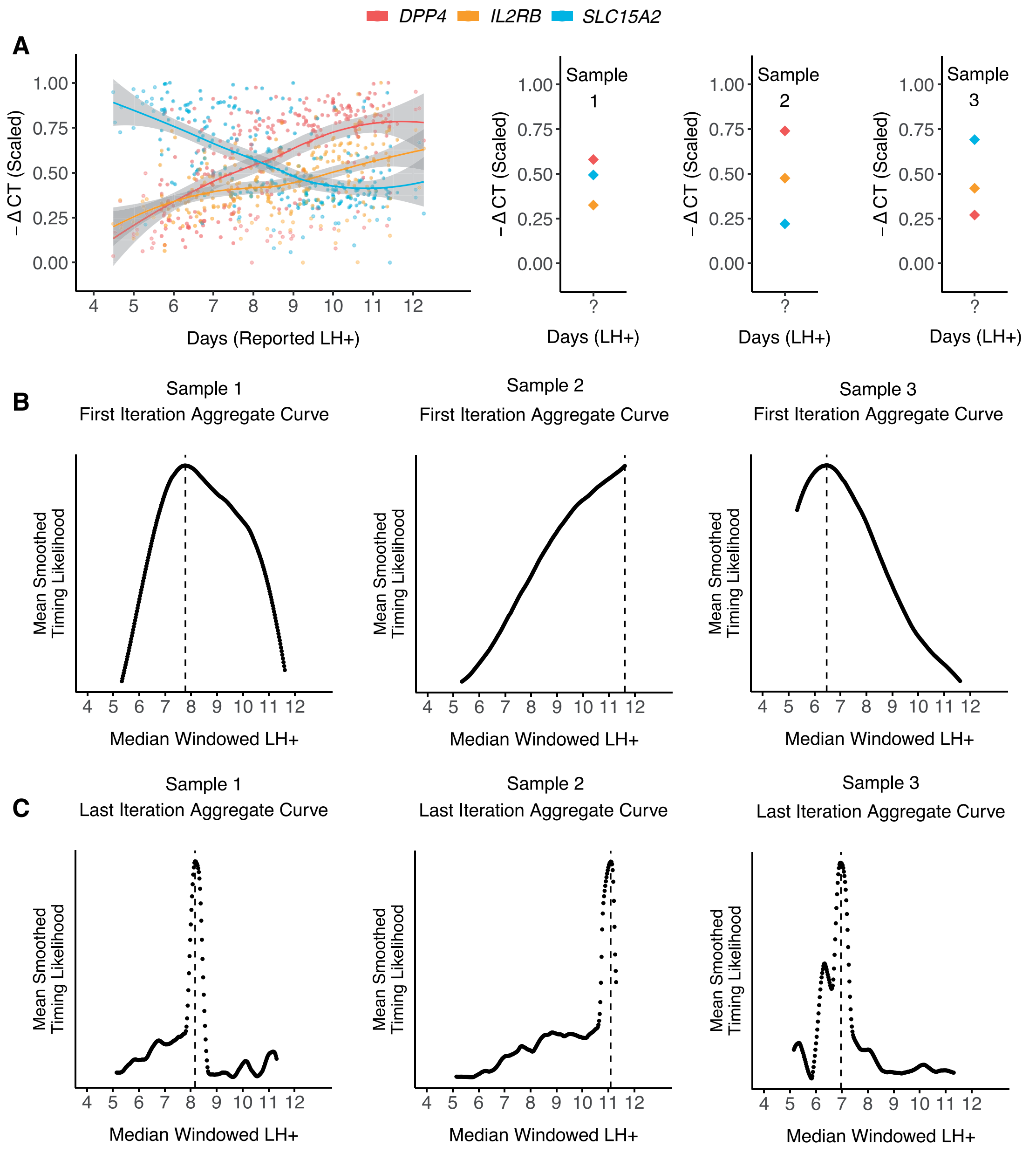

Supplementary Figure S3. Confidence in estimates increases over multiple iterations. (**A**) Computing temporal profiles. Left: Regression curves fit to expression data for three genes of the timing marker panel. Right: Expression values for three samples are the basis for re-evaluating timings of these samples. (**B**) The results of the first iteration of timing estimation for these samples. Each panel gene is used to compute a likelihood that a sample was taken at a particular time point; these are then aggregated into a single likelihood curve per sample, with the peak maxima selected as the temporal estimation for this iteration. (**C**) The results of the final iteration of timing estimation for each selected sample. Modelling has reached a threshold whereby continued iterations are considered to be of negligible benefit; likelihood curve peaks have sharpened substantially over preceding iterations, providing greater confidence in the estimated time value per sample.
