# Supplementary material for: EndoTime: non-categorical timing estimates for luteal endometrium
Source: Hum Reprod. 2022 Jan 29;37(4):747–61. doi: 10.1093/humrep/deac006 (PMC8971653; doi:10.1093/humrep/deac006)
Supplement: deac006_Supplementary_Figure_S4 [file deac006_supplementary_figure_s4.pdf]

Human Reproduction, pp. 1–1, 2022
https://doi.org/10.1093/humrep/deac006

human reproduction

SUPPLEMENTARY DATA

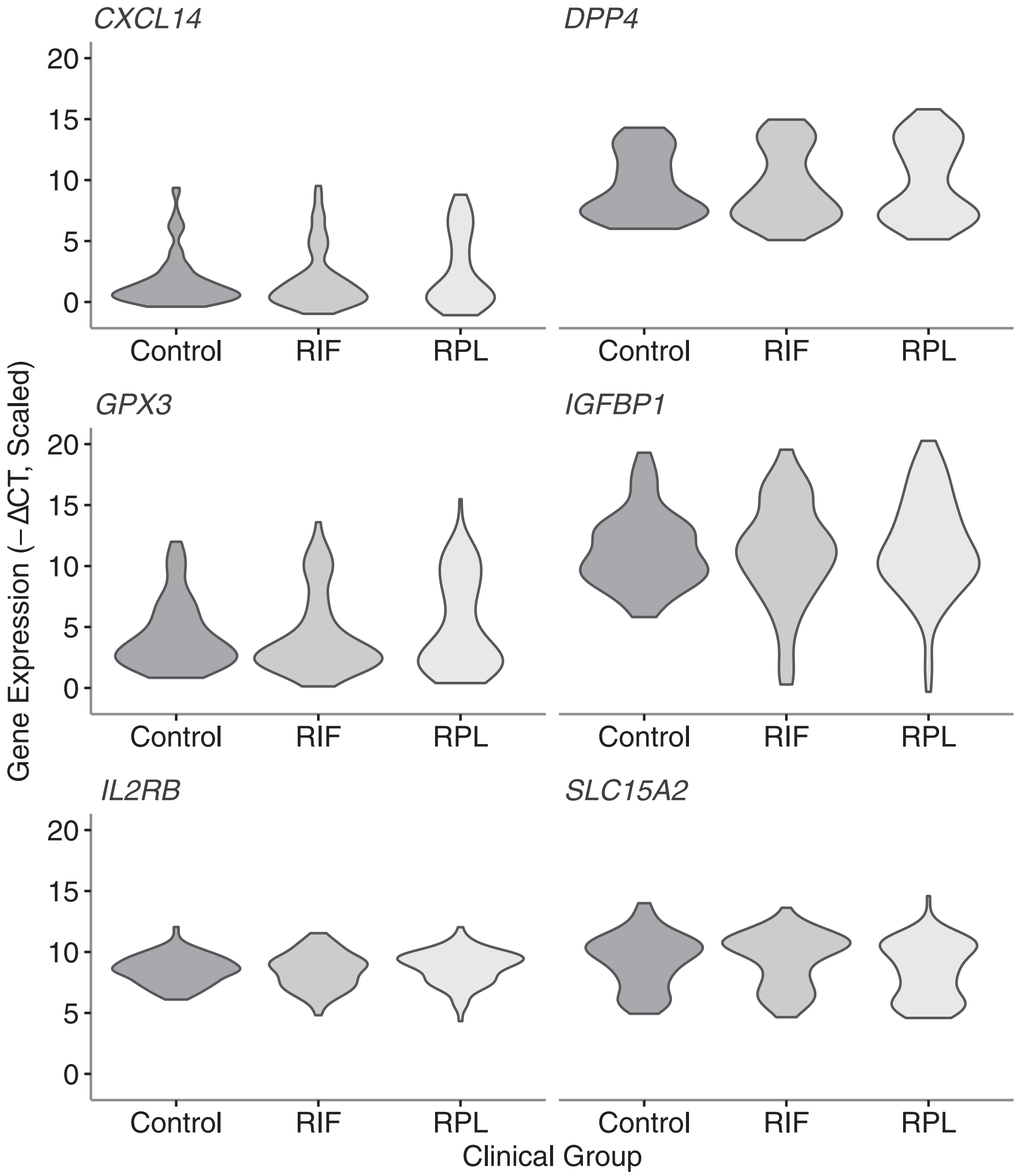

Supplementary Figure S4. Expression distribution in three clinical groups comprising Data Set I for the EndoTime panel genes. Expression values for each gene across the Control (n = 80), RIF (n = 81) and RPL (n = 96) subsets are all sampled from similar distributions, providing confidence that all contribute to modelling in an equitable manner. Pairwise comparisons between clinical groups for each gene: $P > 0.05$, Wilcoxon rank-sum test. RIF, recurrent implantation failure; RPL, recurrent pregnancy loss.
