# Supplementary material for: EndoTime: non-categorical timing estimates for luteal endometrium
Source: Hum Reprod. 2022 Jan 29;37(4):747–61. doi: 10.1093/humrep/deac006 (PMC8971653; doi:10.1093/humrep/deac006)
Supplement: deac006_Supplementary_Figure_S5 [file deac006_supplementary_figure_s5.pdf]

**Human Reproduction, pp. 1–1, 2022**
https://doi.org/10.1093/humrep/deac006

human reproduction

SUPPLEMENTARY DATA

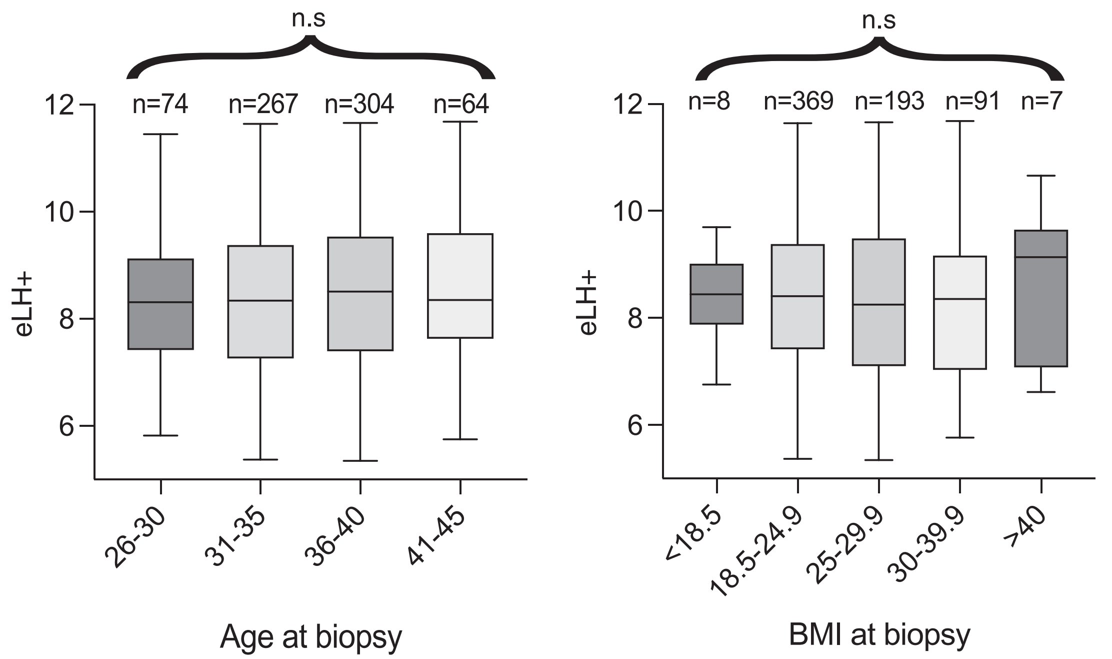


**Supplementary Figure S5. Distribution of EndoTime timing estimates according to patient data.** (**A**) Distribution of estimates by patient age ($P < 0.05$, ordinary one-way ANOVA with Tukey's multiple comparisons test). (**B**) Distribution of estimates by patient BMI ($P < 0.05$, Kruskall–Wallis with Dunn's multiple comparisons test). n.s., non-significant.
