# Supplementary material for: EndoTime: non-categorical timing estimates for luteal endometrium
Source: Hum Reprod. 2022 Jan 29;37(4):747–61. doi: 10.1093/humrep/deac006 (PMC8971653; doi:10.1093/humrep/deac006)
Supplement: deac006_Supplementary_Table_S1 [file deac006_supplementary_table_s1.pdf]

Human Reproduction, pp. 1–1, 2022
https://doi.org/10.1093/humrep/deac006

human reproduction

SUPPLEMENTARY DATA

Supplementary Table SI **Patient demographics for Datasets I, II and III.**

	Dataset I	Dataset II
n	257	36
Age (years) (median (IQR))	36 (33–38)	33 (31–36)*
BMI (median (IQR))	24.0 (22.0–27.8)	24.0 (23.0–29.3)
Previous losses (median (range))	1 (0–18)	4 (0–9)*
Reported LH+ day (median (IQR))	9 (7–10)	8 (7–8)*
Live births [mean (range)]	0.19 (0–2)	0.23 (0–1)

Dataset I	Control	RPL	RIF
N	80	96	81
Age (years) (median (IQR))	36 (33–37)	36 (33–38)	36 (33.5–38)
BMI (median (IQR))	22 (21–26)	25 (22–29)*	24 (21.3–26.7)*
Previous losses (median (range))	0 (0–3)	5 (3–18)*	0 (0–3)
Reported LH+ day (median (IQR))	8 (7–10)	8.5 (7–10)	9 (7–10)
Live births [mean (range)]	0.11 (0–2)	0.37 (0–2)*	0.04 (0–1)

Dataset III	0 losses	1 loss	2 losses	3 losses	4 losses	5 losses
n	54	64	74	74	66	75
Age (years) (median (IQR))	35 (32–38)	36 (33–39)	37 (32–39)	35 (33–38)	35 (33–37)	36 (33–38)
BMI (median (IQR))	22.0 (20.8–26.0)	23.0 (21.0–26.9)	24.0 (22.0–28.0)	24.0 (22.9–26.6)	23.0 (21.0–27.0)	26.5 (22.8–29.5)*
Reported LH+ day (median (IQR))	8 (7–9)	8 (7.8–9)	8 (7–9)	8 (7.8–9)	8 (7–10)	8 (7–9)
Live births [mean (range)]	0.07 (0–1)	0.12 (0–1)	0.28 (0–2)	0.52 (0–4)*	0.49 (0–4)*	0.51 (0–5)*

Data were tested for normality using Shapiro–Wilk test. For Dataset I, *P*-value was calculated by unpaired *t*-test for normally distributed data (age) or Mann–Whitney test for non-normally distributed data (BMI, reported LH+ day and live births). For Datasets II and III, *P*-value against control or 0 losses was calculated by ordinary one-way ANOVA with Dunnett's multiple comparisons test for normally distributed data (age) or Kruskal–Wallis with Dunn's multiple comparisons test for non-normally distributed data (BMI, previous losses, reported LH+ day and live births).

*$P < 0.05$.

IGR, interquartile range; RIF, recurrent implantation failure; RPL, recurrent pregnancy loss.
