# Supplementary material for: EndoTime: non-categorical timing estimates for luteal endometrium
Source: Hum Reprod. 2022 Jan 29;37(4):747–61. doi: 10.1093/humrep/deac006 (PMC8971653; doi:10.1093/humrep/deac006)
Supplement: deac006_Supplementary_Table_S2 [file deac006_supplementary_table_s2.pdf]

Human Reproduction, pp. 1–1, 2022
https://doi.org/10.1093/humrep/deac006

human reproduction

SUPPLEMENTARY DATA

Supplementary Table SII **Distribution of patient-reported LH+ timings in samples sets.**

	Reported LH+ day							
	**5**	**6**	**7**	**8**	**9**	**10**	**11**	**10**
**Dataset I**	13	29	42	43	43	42	40	5
**Dataset II**	0	3	11	16	6	0	0	0
**Dataset III**	4	17	86	118	101	58	15	8
