# Supplementary material for: EndoTime: non-categorical timing estimates for luteal endometrium
Source: Hum Reprod. 2022 Jan 29;37(4):747–61. doi: 10.1093/humrep/deac006 (PMC8971653; doi:10.1093/humrep/deac006)
Supplement: deac006_Supplementary_Table_S3 [file deac006_supplementary_table_s3.pdf]

Human Reproduction, pp. 1–1, 2022
https://doi.org/10.1093/humrep/deac006

human reproduction

SUPPLEMENTARY DATA

Supplementary Table SIII Timing marker genes used for the EndoTime method.

Marker gene		Primary expression domain	Forward primer	Reverse primer
CXCL14	C-X-C Motif Chemokine Ligand 14	Epithelial	5′-AAG GGA CCC AAG ATC CGC TA-3′	5′-GAC ACG CTC TTG GTG GTG AT-3′
DPP4	Dipeptidyl Peptidase 4	Epithelial	5′-CCA AAG ACT GTA CGG GTTC C-3′	5′-ACA AAG AAC TTT ACA GTT GGA TTC AC-3′
GPX3	Glutathione Peroxidase 3	Epithelial	5′-GGG GAC AAG AGA AGT CGA AGA-3′	5′-GCC AGC ATA CTG CTT GAA GG-3′
IGFBP1	Insulin-like Growth Factor Binding Protein 1	Stromal and Epithelial	5′-CGA AGG CTC TCC ATG TCA CCA-3′	5′-TGT CTC CTG CCT TGG CTA AAC-3′
IL2RB	Interleukin 2 Receptor Subunit β	Uterine Natural Killer (uNK) cells	5′-GCG CCT GAC ATT CAC ACA-3′	5′-GCC TAG GAA AGA TAC GTG G-3′
SLC15A2	Solute Carrier Family 15 Member 2	Epithelial	5′-AGG AGG CAT CAA ACC CTG T-3′	5′-CTA GTC CGT TCC TCT GCA TGT-3′
L19	Ribosomal Protein L19	Housekeeping gene	5′-GCG GAA GGG TAC AGC CAA T-3′	5′-GCA GCC GGC GCA AA-3′
