# Supplementary material for: EndoTime: non-categorical timing estimates for luteal endometrium
Source: Hum Reprod. 2022 Jan 29;37(4):747–61. doi: 10.1093/humrep/deac006 (PMC8971653; doi:10.1093/humrep/deac006)
Supplement: deac006_Supplementary_Table_S4 [file deac006_supplementary_table_s4.pdf]

Human Reproduction, pp. 1–1, 2022
https://doi.org/10.1093/humrep/deac006

human reproduction

SUPPLEMENTARY DATA

Supplementary Table SIV Candidate timing marker genes to be used for the EndoTime method.

Candidate marker gene	Reason for interest
ANLN	Putative stem cell marker
AOC3	Gene of interest from a previous project looking at endometrial pericytes
CXCL14	Recruitment of uterine natural killer cells
DIO2	Marker of progesterone resistant decidual subpopulation
DPP4	Potentially involved in the recruitment of bone marrow derived stem cells to the endometrium
EOGT	Gene from a previous project involving linking metabolism, obesity and miscarriage
GPX3	Timing marker
IGFBP1	Classical *in vivo* marker of decidualization
IL15	Activation of uterine natural killer cells
IL2RB	Uterine natural killer cell marker
PER2	Circadian rhythm gene previously associated with a number of previous miscarriages
PRL	Classical *in vivo* marker of decidualization
SCARA5	Marker of progesterone resistant decidual subpopulation
SLC15A2	Timing marker
TIMP3	Decidual expressed gene involved in uterine natural killer clearance of senescent decidual subpopulations
